# The feasibility and acceptability of short-term, individual existential behavioural therapy for informal caregivers of patients recruited in a specialist palliative care unit

**DOI:** 10.1186/s12904-016-0160-1

**Published:** 2016-10-24

**Authors:** Helena S Stöckle, Sigrid Haarmann-Doetkotte, Claudia Bausewein, Martin J Fegg

**Affiliations:** Department of Palliative Medicine, Ludwig-Maximilians-University, Marchioninistr. 15, 81377 Munich, Germany

**Keywords:** Palliative care, Informal caregivers, Caregiver interventions, Feasibility study, Existential behavioural therapy

## Abstract

**Background:**

Existential behavioural therapy (EBT) is a recently developed intervention to support informal caregivers of patients in a specialist palliative care unit and was initially established as a six-session group programme. This pilot study aimed to test the feasibility and acceptability of an adapted short-term, individual approach of EBT in preparation for a randomized controlled trial (RCT).

**Methods:**

The study was conducted in a prospective, mixed methods design including four quantitiative assessments with embedded qualitative interviews at one assessment.

The intervention offered two one-hour therapeutic sessions focusing on (1) mindfulness and (2) existential meaning-in-life as a source of strength provided by a trained psychotherapist. To test the feasibility of the intervention, doubling of the participation rate, compared to the previous group study (13,6 %) as well as an attrition rate of less than 30 % were set as thresholds. To test the acceptability of the intervention, self-rated usefulness of individual aspects of the intervention and the frequency of implementing therapeutic elements by the carers were set as criteria. Acceptability testing also included the number of participants who completed both sessions, where we expected more than 75 % as a criterion for acceptability. Return rates of quantitative questionnaires were set as criteria for the feasibility of data collection (<33 % loss expected within the study period). Qualitative interviews were used to collect additional data on feasibililty and acceptability and to explore potential harms and benefits of the intervention.

**Results:**

44/102 (43,1 %) of eligible informal caregivers agreed to participate in the study. Due to attrition of 13 caregivers (attrition rate: 29,5 %), 31 caregivers were included in the trial. Self-rated usefulness showed sufficiant results for all but one individual aspect. Frequency of implementing therapeutic elements showed wide inter-item as well as inter-participant ranges and decreased over the study period. All participants completed both sessions. Return rates of the questionnaires were within the expected range. According to the interviews, the intervention was associated with several participant-identified benefits. No severe adverse effects were observed.

**Conclusions:**

Findings suggest that the short-term, individual EBT proved feasible and mostly acceptable.

**Electronic supplementary material:**

The online version of this article (doi:10.1186/s12904-016-0160-1) contains supplementary material, which is available to authorized users.

## Background

Informal caregivers of palliative patients are frequently suffering from high burden including depressive symptoms or psychological disorders [1–3]. Informal caregivers bear a considerable lack of wellbeing or quality of life, decreased general health status, anxiety disorders or mood disturbances [3–5]. Additionally, caregivers are confronted with life restrictions regarding social, cultural or occupational issues [5]. Stress levels can be partially higher in carers than in patients themselves [6]. Caregiver’s burden also seems to be significantly impacted by patient’s proximity to death [7–9].

Even if more efforts had been made in the past decades to focus on programmes aiming to support informal caregivers of patients towards the end of life, there is still a need for further research: a systematic review of interventions for informal caregivers in cancer and palliative care reported an overall lack of adequate methodology and underscored the importance for further development of interventional studies [10]. Of 22 studies included in this review, only two interventions were evaluated in a randomised controlled trial (RCT). Although quality and quantity of psychosocial interventions for family carers slightly increased, there is still a need for more research in order to meet the supportive needs of family carers [11]. A Cochrane Review identified evidence that supportive interventions may help to reduce caregivers’ psychological distress but there is further need to explore the identified benefits and to assess interventional effects and potential harms [12].

We have therefore developed existential behavioural therapy (EBT) which is based on existential psychology and the “third wave” of behavioural therapy [13]. The cognitive behavioural therapies have been developed considerably in recent years: concepts such as mindfulness, metacognition, acceptance, personal values, meaning in life and spirituality were integrated into the ‘third wave’ therapies [14]. In the situation of informal caregivers, in their confrontation with the circumstances of existence and the finiteness of life (e.g., inevitablity of death, responsibilites, isolation, freedom, quests for meaning) it is also necessary to integrate concepts of existential psychology [15].

EBT was developed as a manualised group-intervention for informal caregivers, consisting of six group sessions [16]. Therapeutic approaches comprised an introduction into mindfulness and acceptance in the context of dying and bereavement, the activation of personal resources, finding meaning-in-life, reflection of existential questions as well as issues of self-care and stress management.

In the previous RCT, EBT has shown long-term effects on quality of life and psychological symptom reduction of informal caregivers [16]. However, results regarding participation were unsatisfactory (participation rate: 13.6 %), which might result from the group design implicating several weeks of waiting for the next group getting started as well as from the demanding time requirement of six therapeutic sessions (in total, 22 h). To improve the intervention, we have now developed a shortened and individualised EBT intervention. The new programme is designed as an individual instead of a group setting approach and consists of two instead of six supportive sessions.

Mindfulness was practiced throughout all group EBT treatment sessions and can therefore be named as one main therapeutic focus of the group intervention. Furthermore, each session of the group EBT included a discussion about existential issues, such as individual meaning in life, issues arising while facing the death of a beloved one, one’s own confrontation with the end of life as well as activating personal resources to cope with these existential challenges [16]. Therefore, these two issues were selected to be included in the present study while aiming for a reduction of the number of meetings by maintaining the most important and helpful therapeutic aspects.

The selection of standardised questionnaires for feasibility of data collection in the present study was based on the previous RCT [16]. Hereby, only those standardised questionnaires had been finally adopted in this pilot study, which have shown to be affected by the intervention. Standardised questionnaires in this pilot study therefore include the Brief Symptom Inventory (BSI-18) for severity of symptoms [17], the Satisfaction with Life Scale (SWLS) for cognitive aspects of quality of life [18], the WHOQOL-BREF for overall quality of life, [19, 20], the Positive and Negative Affect Scale (PANAS) for affective state [21] and the general health questionnaire (GHQ-12) for psychological distress and functioning [22, 23].

The aim of this study was to test the feasibility and acceptability of this short-term individual EBT approach and to provide information for the planning of an RCT.

The specific objectives were

- to test the feasibility of the intervention with regard to the thresholds for participation and attrition rates;
- to test the acceptability of the intervention with regard to self-rated usefulness of individual therapeutic aspects, frequency of implementing therapeutic elements and number of participants who completed both sessions;
- to test the feasibility of data collection with regard to return rates of questionnaires at four assessments;
- to collect additional data on feasibility and acceptability and to explore potential harms or benefits of the intervention in qualitative interviews.

## Methods

### Design

The study has a prospective mixed methods design, including four quantitiative assessments with embedded qualitative interviews at one assessment.

### Setting and participants

Potential participants were recruited from the Department of Palliative Medicine, Munich University Hospital, Germany. The patients suffered from various diagnoses (mainly advanced cancer, neurological diseases etc.). Potential participants were identified through referrals from staff. Referrals were based on predetermined inclusion and exclusion criteria as well as on individual reasons, such as timely or organisational constraints (e.g., mismatch between caregiver’s availability and working hours of the research staff) and specific caregivers statement (e.g., caregivers asked for privacy/no psychological support). Decisions, whether caregivers were invited for participation were finally made in each individual case by the interdisciplinary team including the resarch staff. In case of uncertainty, caregivers were invited for participation. Recruitment was out-reaching in this study, which means that caregivers who were already seeking help as well as those who did not were asked to participate. Eligible participants were informal caregivers (e.g., family members, friends) of a patient cared for in this specialist palliative care unit during the recruitment period, at least 21 years old and fluent in German. Exclusion criteria were conditions that could adversely affect the ability to participate in the study and to give informed consent (e.g., severe mental disorders like dementia, delirium, acute psychosis). To ensure adherence to predetermined inclusion and exclusion criteria, a research assistant (HS) checked these criteria while recruiting caregivers.

Whereas recruitment was fully conducted during in-patient treatment, some therapeutic sessions had also been delivered after the patient was discharged home or deceased. All therapeutic sessions took place in the premises of the Department of Palliative Medicine.

As this was a feasibility study, comparative analysis was not intended, thus adequate powering of the trial was not required. Based on existing clinical and research experience, we defined a sample size of 30 participants as adequate [24–27].

### Description of the intervention

The intervention consisted of two sessions, each lasting 45 to 60 min. Both therapeutic sessions were provided by a licensed mental health professional (psychotherapist with years of experience in palliative care, Sigrid Haarmann-Doetkotte, SHD) who received training in the theoretical background and practice of the study content by the principal investigator (MF) before and during the entire study. A detailed therapeutic manual provided step-by-step instructions and allowed the therapist to adapt each session to individual needs of participants.

The first session included a mindfulness training, which is based on the concept of mindfulness-based stress reduction (MBSR) [28]. The session started with a short “body scan” to come into the presence. Subsequently, the guided mindfulness meditation began: caregivers were asked to concentrate on their breathing and to return to it whenever their attention wandered while aiming to develop an internal attitude of mindfulness. Hereby, the therapist provided assistance to imagination practice (e.g., imagination of one’s own thoughts as clouds, passing over the sky). After finishing the guided meditation, participants received an audio CD and were specifically encouraged to practice at home at least once a day with the audio CD. Furthermore, they were encouraged to include repetitive tasks of daily life, such as brushing one’s teeth or washing dishes, with an increased level of mindfulness. The audio CD was recorded by the therapist who delivered the session, it’s actual content was a spoken text file (length: 15 min), based on the same template of the therapeutic manual which was used in the session.

The second session aimed to activate caregivers’ personal sources of strength based on their current most meaningful area in life. Initially, participants were asked to determine their current most meaningful life area from which they draw positive and empowering strength, followed by a guided imagination to fully envisage and enhance the positive and empowering emotion related to that area. Participants were invited to name a symbol (e.g., a photograph) representing that source of strength, to be subsequently integrated as an anchor into everyday’s life. For example, if the resource was a pleasant memory represented by a photograph, participants were asked to pin the photograph at a frequently viewed place to increase its present availability.

Based on the previous research experience, we estimated to have a time lag of 7-14 days between the two sessions [16]. However, there was no a-priori threshold regarding the intersessional period.

### Outcomes

To test the feasibility of the intervention, doubling of the participation rate compared to the previous group study (13.6 %), as well as an attrition rate of less than 30 % were set as thresholds.

To test the acceptability of the intervention, self-rated usefulness of individual aspects of the intervention and the frequency of implementing specific therapeutic elements were set as criteria. Furthermore, acceptability testing included the number of participants who completed both sessions, expecting more than 75 % as a criterion for acceptability.

Testing the feasibility of measurement included the return rates of completed questionnaires at different assessment points. Due to the long follow-up period of six months, we expected approximately less than one third of questionnaires to be missing at the 6-month follow-up data collection.

### Outcome measures

The number of caregivers who agreed to participate as well as those who dropped out were noted by the research assistant (HS). Participation rate was the proportion of invited caregivers who agreed to participate by providing informed written consent. Attrition rate was the proportion of informal caregivers who initally agreed to participate but dropped out later.

Regarding acceptability of the intervention, caregivers were asked to complete questionnaires on usefulness and frequency of implementing therapeutic elements. In the questions on the experienced usefulness, participants were asked to rate the usefulness of six components of each intervention, such as the therapist, the mindfulness exercise with paying attention to breathing or determining one’s own sources of strength. Participants rated each component from "0 = not helpful at all" to "4 = extremely helpful". In the questionnaire on the frequencies of implementing therapeutic elements into daily life, participants were asked to state their frequency of implementing particular routines experienced in both therapeutic sessions as free text/per week. The completion of both therapeutic sessions was recorded by the therapist (SHD). To assess the feasibility of measurement, return rates of completed questionnaires were collected by the research assistant (HS).

### Data collection

Quantitative data was collected at four assessments: after caregivers agreed to participate, they were asked to complete the pre-treatment questionnaire (t1), followed by both therapeutic sessions. Subsequenly, the post-treatment data collection (t2) was carried out immediately after finishing the second session. Referring to t2, the follow-up data were collected 4-weeks (t3) and 6-months (t4) later. Participants received the questionnaires for t3 and t4 either in person (during the meeting for qualitative interviews) or by mail, in both cases along with a post-free return envelope.

Standardised questionnaires were used at all four assessments. The pre-treatment data collection furthermore included questions on participants’ demographic characteristics. At the follow-up measures, participants were also asked to self-complete the questionnaires about the experienced usefulness (t3) as well as the frequency of implementing therapeutic elements into their everyday life (t3, t4).

Informal caregivers received an oral explanation by the research assistant regarding the handling of the questionnaires along with the first form and were then asked to self-complete the questionnaires at each assessment point. At any time of the trial, participants were given the opportunity to adress their questions to the research assistant in person, by phone or email. Data was collected (including qualitative interviews) by the research assistant who was not involved into the delivery of the intervention.

### Qualitative interviews

Qualitative interviews were conducted to collect additional information on feasibililty and acceptability and to explore potential harms and benefits of the intervention.

For the qualitative data, semi-structured interviews were conducted with selected participants of the study. A sampling frame was developed to balance participants of qualitative interviews regarding age, gender, occupation and caregiver-patient relationship (Table 1). It comprises characteristics of the population that ideally should be selected in order to ensure a valid selection of the surveyed individuals [29]. The content-related interviews were based on an interview guide, focussing on caregivers’ perception of study structure (consent discussion, therapist, premises/setting of the trial, time requirements, data collection) and the content of both therapeutic sessions. The interview guide is available as Additional file 1. Qualitative interviews were embedded in the 4-weeks follow-up measurement.

**Table 1:** Sampling frame - target and actual values of interviewees

		Target: participants to be interviewed (*n* = 15)	Actual: Participants interviewed in fact (*n* = 15)
Age	<65 years	4-12	11
	>65 years	4-12	4
Sex	female	4-12	8
	male	4-12	7
Caregiver-patient relationship - The patient is my:	wife/husband or partner	4-12	10
	close family member	2-4	4
	close friend	2-4	1
Employed	yes	4-12	11
	no	4-12	4

### Data management and analysis

Results of the usefulness and frequency of practical implementation of therapeutic elements are presented as mean value, standard deviation and range (minimum to maximum).

The intersessional time lags were noted by the therapist (SHD) and are presented as median and range (minimum to maximum time).

The selected questionnaires were also used to determine potential outcome measures in conjunction with obtaining initial estimates (standard deviations) being used for a sample size calculation of the RCT.

Interviews were audio-recorded and subsequently transcribed verbatim by using the transcription software "f5" (https://www.audiotranskription.de/f4.htm). Transcripts were further processed with the qualitative software "MaxQDA" [30]. The methodical evaluation and interpretation of the interviews was based on "summary of content" of the qualitative content analysis [31]. Transcripts were systematically processed by a coding strategy, matching written text fragments to associated subitems of the stated research questions. Coding was conducted by the research assistant (HS) and then reviewed by the principal investigator (MF) to ensure the rigour of the analysis.

The completion of the sampling frame was noted by the research assistant (HS).

## Results

### Recruitment, participation, rejection, and attrition rates

Participants were recruited from 11/2013-09/2014. From a total of 238 patients, 102 eligible caregivers were included in the recruitment. 44/102 eligible informal caregivers initially agreed to participate (participation rate: 43,1 %). Due to an attrition of 13 caregivers (attrition rate: 29,5 %) 31 caregivers were included into the study. 12/13 caregivers dropped out of the study before the baseline questionnaires were completed. One caregiver dropped out between the first and second session.

### Sample

Thirty-one informal caregivers took part in the study. More than two thirds were female, most were married or lived in permanent partnership and two thirds were the spouse or partner of the patient. Table 2 provides a detailed overview on the demographic characteristics of study participants.

**Table 2:** Demographic characteristics of participants (*n* = 31)

Age (years) M ± SD	52,2 ± 13,6
Sex *n* (%)	
female	22 (71,0 %)
male	9 (29,0 %)
Marital status *n* (%)	
married	20 (64,5 %)
permanent partnership	5 (16,1 %)
living alone/single	3 (9,7 %)
divorced/separated	1 (3,2 %)
widowed	2 (6,5 %)
Children *n* (%)	
no	11 (35,5 %)
yes	20 (64,5 %)
Education *n* (%)	
elementary/secondary	3 (9,7 %)
middle/vocational	10 (32,3 %)
grammar school	6 (19,4 %)
university degree	11 (35,5 %)
other	1 (3,2 %)
Employment *n* (%)	
full time (>35 h)	13 (41,9 %)
part time (<35 h)	6 (19,4 %)
houseman/housewife	3 (9,7 %)
retired	6 (19,4 %)
other	3 (9,7 %)
Religion *n* (%)	
protestant	9 (29,0 %)
catholic	9 (29,0 %)
orthodox	1 (3,2 %)
none	12 (28,7 %)
Relation - patient is *n* (%)	
husband/wife	13 (41,9 %)
unmarried partner	4 (12,9 %)
parent	10 (32,3 %)
child	1 (3,2 %)
other	3 (9,7 %)

### Time lag between both sessions

Results of this pilot study showed that time lags between both sessions were within a range from one to 50 days with a median of 7 days. Inter-participant variations of time lags between the two sessions were caused by both, personal caregiver issues (e.g., patient’s condition detoriated and caregivers wished to postpone the second session) or because the therapist was not available at the required dates.

### Acceptability of the intervention

The self-rated usefulness showed sufficiant results for the single items of both therapeutic sessions (Table 3). Only the self-reliant audio CD practice in daily life was less well received. The frequency of implementing therapeutic elements in daily routines showed wide inter-item and inter-participant ranges (Table 4). Overall, the recommended aim of practicing with the audio CD at least once a day was not met by most of the participants and the frequency of practice showed a decrease in all four items from t3 to t4 (Table 4). Again, self-reliant practice with the audio CD was the least-practiced routine (Table 4). Being fully mindful with a repetitive task of daily life was practiced comparatively often by participants. All participants completed both therapeutic sessions.

**Table 3:** Helpfulness of individual elements of the therapeutic sessions (0 = not helpful at all; 4 = extremely helpful)

First Session - mindfulness	Mean (SD) range
Therapist	2,9 (1,1) 0-4
Information/Psychoeducation	2,9 (1,0) 0-4
Body-Scan (Introduction)	2,6 (1,1) 0-4
Mindfulness exercise (paying attention to the breath)	2,9 (1,1) 0-4
Mindfulness imagination (e.g., imagination of one’s own thoughts as clouds, passing over the sky)	2,5 (1,0) 0-4
Self-reliant practice with audio CD	2,0 (1,1) 0-4
Second Session - Resources	
Therapist	3,0 (1,1) 1-4
Information/Psychoeducation	2,9 (1,1) 0-4
Determining the sources of strength	3,0 (1,0) 1-4
Guided imagination of resources and their empowering emotion	2,9 (1,0) 1-4
Finding a symbol (representing the resource)	2,8 (1,1) 1-4
Self-reliant handling of resources/symbols in daily life	2,8 (1,1) 1-4

**Table 4:** Frequency of practical implementation of particular routines learned in both therapeutic sessions (free text/per week)

4-week follow-up	Mean (SD) range
Self-reliant practice with audio CD	1,9 (1,5) 1-7
Being fully mindful with a repetitive task of daily life	4,9 (4,3) 1-15
Using the selected symbol for envisioning one’s personal source of strength	4,2 (4,3) 1-15
Experiencing an empowering and warming sensation while practicing	4,2 (3,9) 1-15
6-months follow-up	
Self-reliant practice with audio CD	1,8 (1,5) 1-6
Being fully mindful with a repetitive task of daily life	3,8 (3,1) 1-14
Using the selected symbol for envisioning one’s personal source of strength	2,5 (2,3) 1-7
Experiencing an empowering and warming sensation while practicing	3,6 (4,4) 1-18

### Feasibility of data collection

Table 5 shows the results regarding the return rates of completed questionnaires at four assessment points. Return rates consist of the number of returned completed questionnaires in proportion to the number of distributed questionnaires.

**Table 5:** Return rates of completed questionnaires at four assessments

Assessment	Completed questionaires (returned/distributed)
pre-treatment (t1)	31/31
post-treatment (t2)	31/31
4-weeks follow-up (t3)	28/31
6-months follow-up (t4)	23/31

### Qualitative interviews

Fifteen interviews with selected participants were conducted (see Table 1: Sampling frame). Eight interviewees were female, eleven younger than 65 years. Ten were wife/husband or partner of the patient, four were family members and one caregiver was a friend of the patient. Eleven interviewees were in paid employment. Interviews lasted on average 34 min (range 17 to 65 min). All interviews were conducted at a place of convenience for the participants, which was in most cases at the hospital or at home of the informal caregivers.

#### Study structure

The majority of interviewees described the aspects of consent discussion, mental health professional and premises or setting of the trial as adequate. The time requirement for the intervention was seen as appropriate by 12/15 interviewees and from their point of view they had no unmet needs for professional support after the second session. The other three interviewees found the time of the intervention too short and they would have wished to have more support, e.g., by having the possibility to make another appointment with the therapist. None of them estimated the time of the intervention as too long. Even if some interviewees found it challenging to concentrate on the questionnaires during difficult times, the scope and content alignment of questionnaires was considered to be reasonable by 12/15 interviewees. There were no reports of serious problems such as inappropriate data collection forms or unrealistic expenditure of time regarding the participation in the study.

#### Mindfulness

The experience of mindfulness appeared to be beneficial for interviewees in various respects. Mindfulness practice was described as a helpful approach to feel inwardly strengthened in difficult situtations by focusing on the present moment and the continuous flow of one’s own breath. Even if burdensome issues did not disappear, interviewees were able to create more inner distance in this respect through practicing. Mindfulness was also seen as a supportive tool in order to reach an inner state of calm and peace of mind and to be inwardly open to anxiety inducing issues. Furthermore, interviewees reported of improvements linked to mindfulness with regard to quality of sleep, concentration, awareness of the presence, gratitude towards life, energy levels or physical complaints such as neurodermatitis.

*The session was a rewarding experience: I could find calm for the first time after weeks and months of trouble. The patient’s condition was very unstable recently. I felt extremely stressed and I did never know what to expect for the following day. I was not able to find a moment of rest and tranquility. But then in just one hour sitting with the therapist to practice the mindfulness meditation, I was able to take a deep breath and I could let go of the troubled things out of this room while simply taking part in this session. (wife, 49 years)*

On the other hand, it seemed not always easy for interviewees to focus on the mindfulness practice while having a busy schedule or when worries took over. Some caregivers expressed the wish for additional support, such as having the possibility to make another appointment with the therapist. At this point, they would have liked to deepen the contents delivered in the mindfulness session or to adress additional individual topics to the therapist.

*It is sad, but I did the practice only twice so far. It was just too much going on around me. There was no time to care for myself. I couldn’t find some time to sit down to practice. I would have wished to have little more support in this context, maybe by coming to another meeting. Whenever I was able to do the practice, I could benefit a lot of mindfulness: It reduced the burden of my sorrows, because I know now, these sorrows are just thoughts, which come and go. (wife, 45 years)*

#### Personal sources of strength

All interviewees instantly gave a specific example of one’s own empowering resource and everyone was able to identify a specific symbol related to it. Resources included intimate relationships/other people, touching memories, creative working or leisure activities, or spirituality. As symbols, caregivers chose various photographs, letters, stones, or a piece of paper with some notes as well as several tangible goods. Focusing on personal sources of strength appeared to be helpful for interviewees in various aspects. While focusing on their resource, interviewees reported to experience a feeling of empowerment on the ability to better cope with troubled situations. By using the symbols as tools in their everyday life, interviewees found it easier to have access to and to feel their positive and strenghtening emotions and energy. Even though the resources were not new to them, interviewees felt their positive impacts to be concretely enhanced through the session.

*In the second session, it was interesting, I considered so much, but then I had this "aha experience" with the sailboat. That is my magnificent memory and my energy. It was mine for years but now I took it up again. I constantly look now at this photograph, which I selected as a symbol. Hereby, I feel so pleased. My last sailing holiday is eight or nine years ago, but I can still feel its positive energy at these moments. Meanwhile, I just close my eyes and the picture appears. The session gave that new impulse. That is something just for me. (husband, 46 years)*

One caregiver decided to choose a symbol, which was associated with the loss of a previously helpful resource. As a consequence, the more sad aspects of this memory emerged. The advice of the therapist not to use this symbol for daily life but to look for another empowering resource was found to be helpful with no additional intervention being needed.

## Discussion

Results of this pilot study showed that criteria set for feasibility of the intervention had been met. The present study achieved more than a doubling of the participation rate compared to the previous group EBT [16]. Attrition rate was in the expected range. Based on this data, the intervention seems to be feasible.

Results on acceptability of the intervention vary: regarding the experienced usefulness of the individual EBT, the items were similarly well rated, except for the item "self-reliant practice with audio CD in daily life", which was seen as comparatively less helpful. Data on the frequency of practical implementation of particular routines showed a decline in exercise from first to second follow-up measurement in every single item, which might result from the stated lack of sufficient ongoing support. The self-reliant practice with the audio CD was the comparatively least practiced routine and did not meet the recommended goal of practicing at least once per day. Harding and Higginson found that caregivers were highly ambivalent with regard to their own needs [32]. In this context, it has to be considered that the audio CD was the comparatively most time consuming practice, which might have had a negative impact on frequency and therefore usefulness of practice. Furthermore, interviewees reported the wish to have more ongoing support, which can also be seen as a possible key to achieve improvement of these results. Interviewees who used the CD did not report of any severe problems concerning content issues of the CD, suggesting that there is no need for reevaluating the CD itself. However, future research is needed to reevaluate the reasonable use of the audio CD while discussing structural amendments or alternative options as appropriate therapeutic tools. Results on the completion of both therapeutic sessions were in the expected range and with regard to this point, the intervention can be found to be acceptable.

Return rates of questionnaires were set as criteria for feasibility of data collection. Return rates of questionnaires were in the expected range at all four assessments. It can therefore be concluded that data colletion proved feasible in this pilot study.

Feedback from the interviews regarding the study’s structure was encouraging, suggesting that there is no need for amendments regarding the reviewed aspects of consent discussion, mental health professional, premises or setting of the trial, time requirement and data collection.

Mindfulness appeared to be helpful in many different ways. In the literature, higher levels of mindfulness have been found to be associated with a greater sense of life satisfaction, better quality of life as well as with less distressing symptoms; the level of mindfulness was described as a predictor of positive developments in caregiver burden [33]. McBee reported that informal caregivers were less stressed and had less physical strains after attending a mindfulness-session [34]. Mindfulness was also found to be significantly correlated with higher levels of well-being, less distress and depression as well as with a higher sense of self-efficacy in informal cargivers [35, 36]. Despite several positive changes in our study, some caregivers found it difficult to continue their practice when circumstances worsened, which again raises the question for the need of additional support. It also has to be considered that mindfulness might not work for every person and situation in the same way. Even if mindfulness is explicitely recommended to informal caregivers by some authors, others also voiced their concerns [34, 37, 38]. Future studies are needed to assess how much additional support would be practicable and beneficial to ease the mindfulness practice for informal caregivers even in difficult times.

Focusing on individual meaning in life and personal sources of strength has been described as important by interviewees to increase one’s ability to cope with critical life events. All interviewees easily coped with the content of the second session and its practical implementation. On this occasion, the findings on existential meaning in life are consistent with other studies: Informal caregivers who focused more on personal resources in the sense of a more meaningful life have been found to be significantly less distressed and reported a better quality of life [16]. Evidence has accumulated that strengthening personal resources by focusing on positive emotions is very important to cope successfully with stressful situations [39]. This study sample included one participant, whose selected symbol respectively the resource was partially associated with grief. The participant was able to limit potential negative consequences by following the advice of the therapist not to use this symbol in daily life. This case, however, demonstrates the importance to focus on present (but not previously) empowering resources.

This pilot study was based on recommendations for good practice for design and analysis of pilot studies, which are used specifically to plan an RCT [40]. According to this, all specifications have been adhered to in this pilot study, except for the randomisation procedure.

### Limitations

First, potential participants were identified through referrals from the staff of the unit. This selection process might have introduced selection bias and study participants might not be representative. To reduce selection bias, every individual case was discussed in the daily interdisciplinary meeting of the unit, including the research staff. In case of uncertainty, caregivers were invited to participate in the study. Second, we did not collect data on the patient’s condition respectively the stage of life-limiting disease or time of death and therefore results cannot be linked to that. Some outcomes were measured while the patient was alive and others at various times in bereavement. This is a major limitation of the study as the patient’s condition, the stage of life-limiting disease or death of the patient may have significantly influenced the response of informal caregivers. The RCT will include this information. Third, we did not include a control group in this pilot study: recruitment, retention and attrition rates as well as the questionnaire completion rate may be different in a randomized study with a control group design. Furthermore, we do not have information about the acceptance of caregivers to be randomly assigned to either the intervention or the control group. However, based on our previous findings [16], we hopefully will reach comparable acceptance rates as in the EBT group trial. Fourth, the intersessional time space was not clearly defined prior to the study. Even if most intervals were between the estimated 7-14 days, the results showed, to some extent, inter-participant variations. Therefore, interactions between study results and different intersessional time lags cannot be ruled out. To reduce this limitation, intersessional time spaces will be clearly defined in future.

## Conclusion

In conclusion, this pilot study has shown that the newly developed short-term, individual EBT for informal caregivers of patients, recruited in a specialist palliative care unit proved to be feasible. Thresholds set for feasibility of intervention and feasibility of data collection were fully met. The intervention showed to be acceptable in most aspects; future research is needed to focus on adjustments with regard to the less well received aspects of the intervention. Qualitative data provides references on potential benefits of the intervention. No severe adverse effects were reported in the interviews. Although facing some limitations, the findings of this pilot study will be used to design a fully powered RCT.

## Additional file

Additional file 1: Guide for the qualitative interviews. (DOCX 29 kb)
