# Supplementary material for: The feasibility and acceptability of short-term, individual existential behavioural therapy for informal caregivers of patients recruited in a specialist palliative care unit
Source: BMC Palliat Care. 2016 Oct 24;15:88. doi: 10.1186/s12904-016-0160-1 (PMC5078917; doi:10.1186/s12904-016-0160-1)
Supplement: Additional file 1: — Guide for the qualitative interviews. (DOCX 29 kb) [file 12904_2016_160_MOESM1_ESM.docx]

**Interview guide**

**ID: __________________________________**

**Name: _______________________________**

**Date: ________________________________**

| **Main questions** | **Relevant topics** | **Additional information** |
| --- | --- | --- |
| How was your experience participating in the study? What was helpful, what barriers did you experience? | How did you experience the information about the study? Did you receive all necessary information? Did you have enough time to ask questions?  How did you experience the therapist?  How did you experience the interventional setting?  How were your experiences with the time frame of the study as a whole / with the interventional sessions?  What do you think about the questionnaire? Were all relevant issues properly addressed?  Was one interventional session better / more enjoyable / more meaningful than the other? | Why?  What exactly?  What were the problems?  What was particularly good?  Did you have the feeling of being well supported? Did you feel being taken seriously?  What were the problems?  What was particularly good?  Was the place for the meeting chosen appropriately? Did you feel protected, being in an intimate atmosphere?  Why?  What exactly?  What were the problems?  What was particularly good? |
| How did you experience the first session on mindfulness? What did you like most, what did you like least? | Was the definition of mindfulness comprehensive and understandable?  Was the theoretical information at the beginning of the session helpful?  Was the exercise at the beginning of the session helpful to experience your body (body scan)?  Did the mindfulness exercises help you to distance yourself from stressful thoughts and feelings?  How did you experience the CD for your home practice? How many times have you performed the exercise / in which situations? What was good with the CD, what was not so good? What would you like to get improved?  Has anything changed in your life through the experience of mindfulness and / or mindfulness practice?  What would you like to add about mindfulness? | Why?  What exactly?  What were the problems?  What was particularly good? |
| How did you experience the second session on meaningful areas in your life? What did you like most, what did you like least? | Was the definition of "resources" comprehensive and understandable?  Has it been easy to figure out your personal sources of power?  Did the intervention encourage you to repeatedly imagine your source of power using the symbol? If so, have you experienced a feeling of strengthening?  Were you able to integrate the exercise into your daily life? Has it changed something in your life?  What would you like to add about the session on meaningful areas in your life? | Why?  What exactly?  What were the problems?  What was particularly good? |
| Looking back: What would you suggest to improve in total or do differently? | Are there any parts of the study you would have had the wish for more, for less?  Why did you decide to participate in the study?  What expectations did you have regarding the study? Were your expectations met?  Would you participate again? Why not?  Whom would you recommend to participate in the study? Whom rather not?  Have you used any other support services?  What would you like to add / get rid of? | Which?  Why?  What exactly?  If yes, which?  Was the support experienced as helpful? |
